# A hepatic sparganosis misdiagnosed as intrahepatic mass-forming cholangiocarcinoma: a case report and literature review

**DOI:** 10.3389/fonc.2024.1391256

**Published:** 2024-04-10

**Authors:** Yinjiao Wang, Yu Lou, Lang Chen, Xin Yang, Aihong Cao, Peng Du

**Affiliations:** ^1^ Department of Radiology, The Second Affiliated Hospital of Xuzhou Medical University, Xuzhou, China; ^2^ Department of Radiology, The 71st Group Army Hospital of the People’s Liberation Army of China, Xuzhou, China

**Keywords:** hepatic sparganosis, cholangiocarcinoma, intrahepatic, tumor, *Taenia mansoni*

## Abstract

Hepatic sparganosis (HS) is extremely rare and has not been previously reported in Eastern China. We report the diagnosis and treatment of a patient with HS from Xuzhou City, Jiangsu Province, China. The patient was admitted due to an acute biliary tract infection, and the symptoms improved after treatment at the Gastroenterology Department. During an ultrasound examination on admission, an abnormal echo was incidentally discovered at the junction of the left and right lobes of the liver. Thereafter, upper abdominal computed tomography (CT) and magnetic resonance imaging (MRI) non-contrast and contrast-enhanced examinations, and serum tumor biomarker examination were completed. After a multidisciplinary treatment (MDT) discussion at the Department of Hepatobiliary Surgery, the patient was diagnosed with intrahepatic mass-type cholangiocarcinoma (IMCC) and surgery was recommended. The patient underwent surgical treatment, and postoperative pathology revealed HS. No signs of intrahepatic recurrence were observed during the 1-year follow-up period.

## Introduction

Sparganosis is a parasitic infectious disease caused by *Taenia mansoni* larvae in the human body (1). The most common parasitic site is the subcutaneous, followed by the eyes, maxillofacial, and central nervous system (2, 3), whereas the parasitic site on the liver is very rare and has not been previously reported in Eastern China. We report on the diagnosis and treatment processes of a patient with HS from Xuzhou City, Jiangsu Province, and a combined literature review analysis to provide a reference for the diagnosis and treatment of this disease.

## Case description

A 51-year-old female patient from Xuzhou City, Jiangsu Province, visited our hospital on December 10, 2022, because of repeated abdominal pain with fever for more than 10 years. Ten days ago, the patient developed right upper abdominal pain without any cause, which showed persistence, paroxysmal aggravation, and the abdominal pain symptom was aggravated after eating, accompanied by fever, and intolerance of cold, and the maximum body temperature was 39.0°C. The patient was otherwise healthy, with no history of infectious disease, surgery, blood transfusion, drug or food allergies, or family history.

Blood routine on admission: percentage of eosinophils, 10.40%↑; absolute value of eosinophils, 0.56 10^9^/L↑; C-reactive protein, 52.80 mg/L↑; liver function: glutamyl transferase, 2290 U/L↑; alkaline phosphatase, 572 U/L↑; pre-albumin, 150 mg/L↑; total bilirubin, 23.0 µmol/L↑, and no abnormalities were present in the remaining items. Upper abdominal ultrasonography revealed multiple calculi in the gallbladder and common bile duct, coarse hair on the gallbladder wall, and a heterogeneous hypoechoic nodule at the junction of the left and right lobes of the liver. The physician considered that the abdominal pain was induced by the gallbladder, common bile duct calculi, and inflammation. Consequently, the patient underwent endoscopic retrograde cholangiopancreatography, endoscopic duodenal sphincterotomy, endoscopic balloon dilation, bile duct calculus lithotripsy, removal, laparoscopic cholecystectomy, and biopsy. The abdominal pain was relieved after treatment.

**Figure 1:**
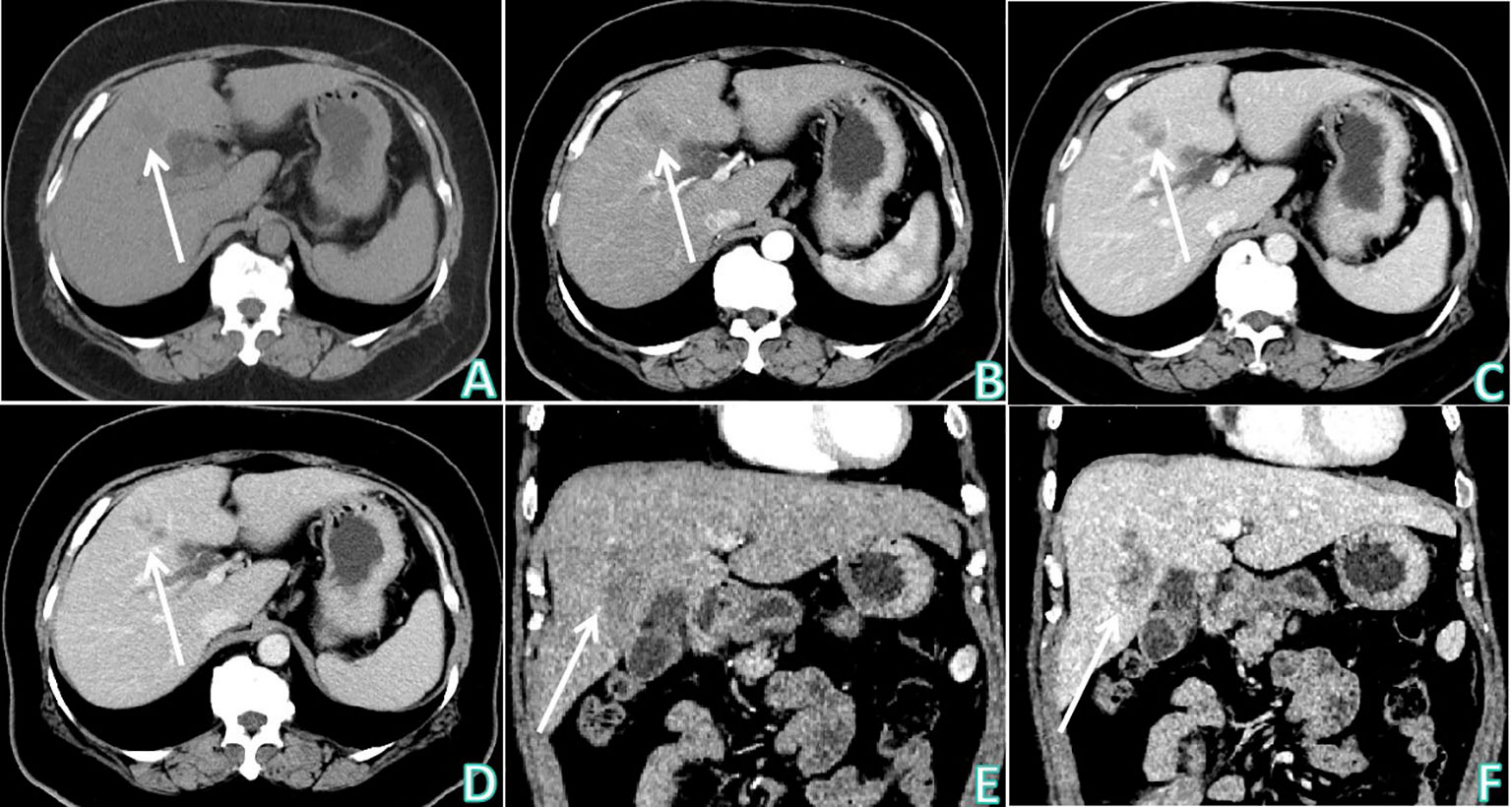
Upper abdominal CT non-contrast and contrast enhanced examinations. **(A)** Upper abdominal CT non-contrast examination (axial) showed: there was an irregular mass-type low density lesion at the junction of the left and right lobes of the liver, which was ill-defined, with uneven density in the interior. **(B–D)** Sequentially, the arterial phase, venous phase, and delayed phase of upper abdominal CT contrast enhanced examination (axial) showed: the enhancement degrees of the lesion at the arterial phase and portal vein phase were lower than the normal liver parenchyma, lamellar zones without enhancement were observed inside the lesion, and edge enhancement was obvious at the delayed phase. **(E)** The arterial phase of upper abdominal CT contrast enhanced examination (coronal). **(F)** The delayed phase of upper abdominal CT contrast enhanced examination (coronal). White arrows indicates that the lesion was observed at the junction of the left and right lobes of the liver.

**Figure 2:**
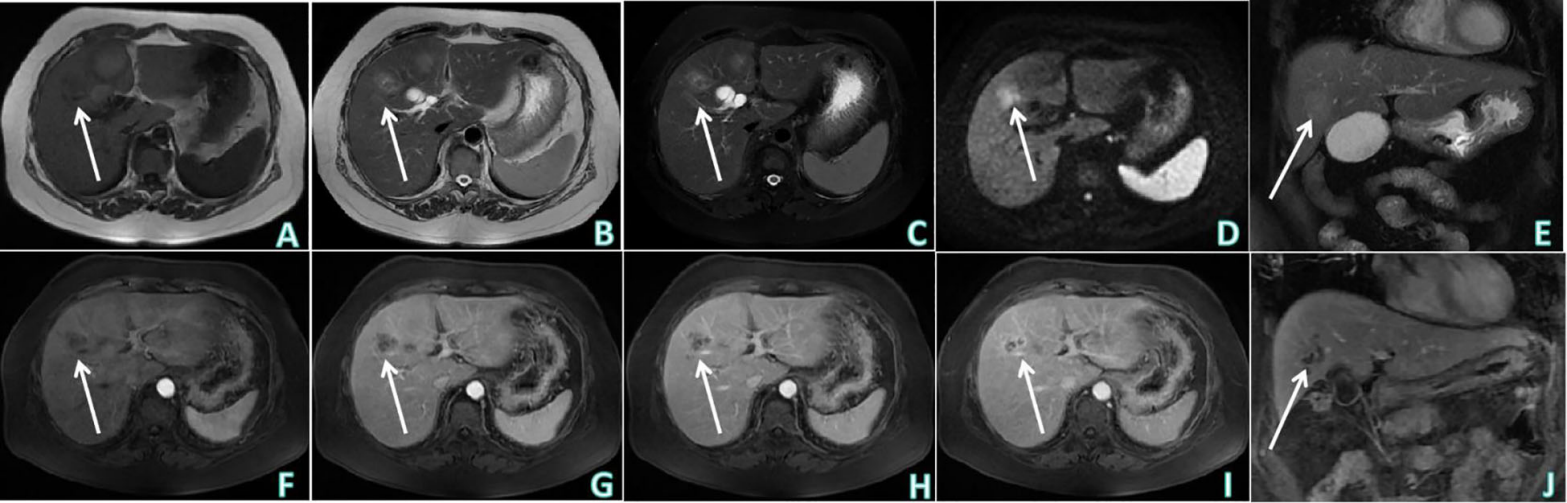
Upper abdominal MRI non-contrast and contrast enhanced examinations. **(A)** T1WI of upper abdominal MRI non-contrast examination (axial) showed: hypointensity of the lesion at the junction of left and right lobes of the liver. **(B, C)** Sequentially, T2WI and T2-SPIR of upper abdominal MRI non-contrast examination (axial) showed: uneven hyperintensity. **(D)** DWI of upper abdominal MRI non-contrast examination (axial) showed: mild diffusion limitation. **(E)** T2-SPIR of upper abdominal MRI non-contrast examination (coronal). **(F–I)** Sequentially, the arterial phase, venous phase, equilibrium phase and delayed phase of upper abdominal MRI contrast enhanced examination (axial) showed: no obvious enhancement was observed on the strip-like hypointensity opacity inside the lesion, the lesion edge showed progressive enhancement. **(J)** The delayed phase of upper abdominal MRI contrast enhanced examination (coronal). White arrows indicates that the lesion was observed at the junction of the left and right lobes of the liver.

The patient underwent non-contrast and contrast-enhanced upper abdominal CT to further determine the nature of the heterogeneous hypoechoic nodule at the junction of the left and right lobes of the liver revealed by ultrasound examination (**Figure 1**). An irregular mass-type low-density lesion was observed at the junction of the left and right lobes of the liver, which was ill-defined with an uneven density in the interior. On enhancement scanning, the enhancement degrees of the lesion in the arterial and portal vein phases were lower than those of the normal liver parenchyma, lamellar zones without enhancement were observed inside the lesion, and edge enhancement was evident in the delayed phase. Subsequently, upper abdominal MRI non-contrast and contrast-enhanced examinations were performed (**Figure 2**), which revealed hypointensity of the lesion at the junction of the left and right lobes of the liver on T1-weighted imaging (T1WI), uneven hyperintensity on T2-weighted imaging (T2WI) and T2-spectral presaturation with inversion recovery (T2-SPIR), and mild diffusion limitation on diffusion-weighted imaging (DWI). However, no enhancement was observed on the strip-like hypointensity opacity inside the lesion, the lesion edge demonstrated progressive enhancement, and a lymph node opacity with a short diameter of approximately 10 mm was observed in the hepatogastric space. The patient’s serum carbohydrate antigen 199 (CA199) of the patient was 335.00 U/mL↑. Based on radiological findings and CA199 examination results, the patient was finally diagnosed with IMCC after an MDT discussion at the Department of Hepatobiliary Surgery, and surgical treatment was recommended.

**Figure 3:**
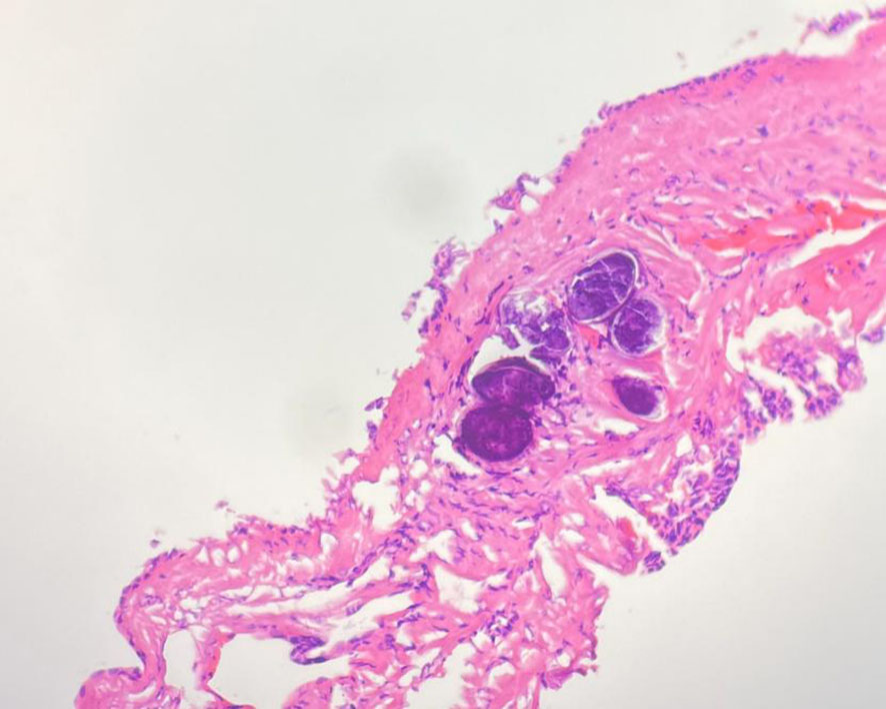
Pathology HE chart showed: chronic inflammation in the liver tissue, with irregular necrotic nodules locally and formation of granuloma in the surrounding epithelium, accompanied by infiltration of lymphocytes, plasmocytes and eosinophils. In addition, a small lesion of highly disintegrated polypide was seen in the necrotic nodule, conforming to parasitic disease.

**Figure 4:**
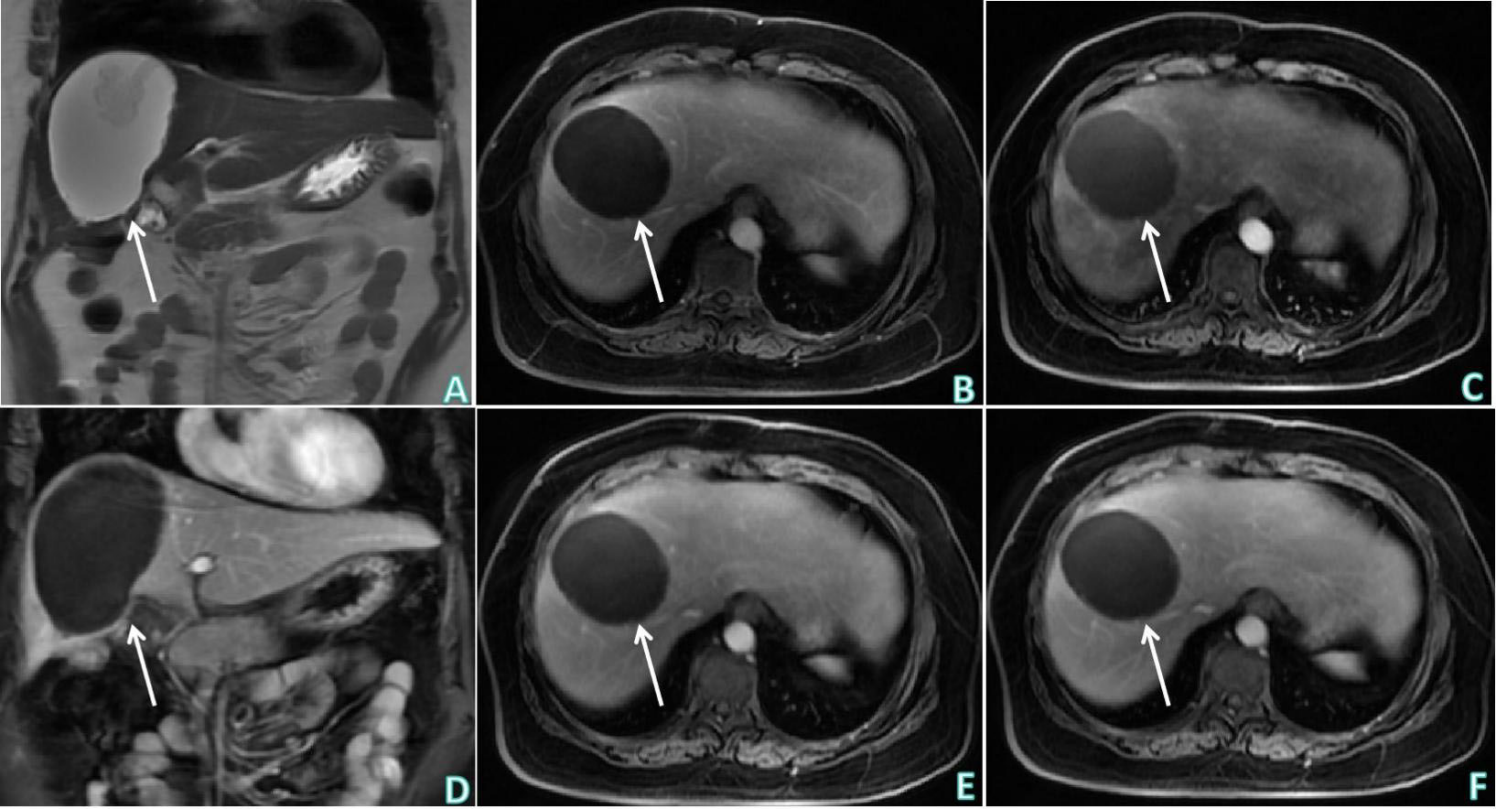
Upper abdominal postoperative MRI non-contrast and contrast enhanced examinations. **(A)** T2WI of upper abdominal MRI non-contrast examination (coronal) showed: postoperative changes of the liver, flake-like hypointensity in the operation area. **(B–F)** Sequentially, the early arterial phase (axial), late arterial phase (axial), delayed phase (coronal), venous phase and equilibrium phase (axial) of upper abdominal MRI contrast enhanced examination showed: postoperative changes of the liver, flake-like non-enhanced hypointensity in the operation area, and no obviously abnormal enhancement in the remaining liver parenchyma. White arrows indicates postoperative changes in the liver, flake-like non-enhanced hypointensity in the operative area.

The patient underwent a middle liver mass resection on December 15, 2022. Intraoperative findings revealed that a part of liver tissue was resected in a page-like manner, a mass (size, 3 × 1.8 × 1.5 cm) was observed on the section, the section of the mass was multi-nodular, gray–white, solid, hard in texture, and well-defined. The liver mass was sent for pathological examination (**Figure 3**), which revealed chronic inflammation in the liver tissue, with local irregular necrotic nodules and the formation of granulomas in the surrounding epithelium, accompanied by the infiltration of lymphocytes, plasmocytes, and eosinophils. In addition, a small lesion of highly disintegrated polypide was observed in the necrotic nodule, consistent with parasitic disease. The patient’s history was recorded, and she informed the physicians that she liked to eat bullfrogs. Blood samples were collected for parasite antibody tests, and the results were positive for the *Sparganosis mansoni* IgG antibody. Combined with the radiological findings, laboratory examinations, and postoperative pathology, the intrahepatic lesion was finally diagnosed as HS. The patients did not receive any anti-inflammatory treatment after surgery. Six months later, the patient did not complain of any discomfort and there were no abnormalities in routine blood tests, liver function, or tumor markers. Upper abdominal ultrasonography revealed postoperative changes in the liver; there was no echoic zone in the operative area and no abnormality was observed in the remaining liver parenchyma. On re-examination 1 year later, the patient did not complain of any discomfort, and there were no abnormalities in routine blood tests, liver function, or tumor markers. Non-contrast and contrast-enhanced upper abdominal MRI (**Figure 4**) revealed postoperative changes in the liver, flake-like non-enhanced hypointensity in the operative area, and no abnormal enhancement in the remaining liver parenchyma.

## Discussions

Sparganosis is a rare amphixenosis that is prevalent in Southeast Asia, Africa, Australia, and the United States (1–3). Cases, mostly sporadic, have been reported in different areas of the world, and in China, the disease is more common in southeastern coastal areas, such as Zhejiang, Guangdong, and Fujian Provinces (4). Sparganosis is an infectious disease caused by *S. mansoni* larvae that parasitize the human body. The life history of *S. mansoni* includes one definitive host and two intermediate hosts, in which the definitive host is mainly cats and dogs, the first intermediate host is cyclops, and the second intermediate host is largely frogs. Multiple vertebrates such as snakes, birds, and pigs can transport hosts. *Spirometra mansoni* is a zoonotic parasite that occasionally infects and parasitizes humans, serving as the second intermediate host, transport host, or definitive host (5). Individuals can get infected in the following manner (1): the pleroceroid invades the wound when the meat and skin of frogs or snakes are used for topical application on the wound (2), eating uncooked or semi-uncooked frogs, snakes, birds, and pork containing *S. mansoni*, and (3) mistakenly swallowing the infected first intermediate host cyclops when drinking uncooked water or swimming (6). Pleroceroids usually maintain larval morphology in the human body, migrate, and parasitize all tissues in the body, mostly under the skin, followed by the eyes, oromaxillofacial region, and central nervous system. The liver is an extremely rare parasitic site (7). Diagnosis of sparganosis is mainly dependent on a combination of immunological, radiological, and pathological examinations. Typically, serum pleroceroid antibody detection is a relatively reliable diagnostic method for this disease (8), and most cases can be confirmed through the discovery of polyps during surgery or tissue biopsy.

In this case, the patient visited our hospital because of gallbladder and bile duct calculi accompanied by an acute biliary system infection. Radiological examinations incidentally revealed an intrahepatic mass, which was confirmed as HS after surgical pathological and serological examinations. Considering the dietary characteristics of eating bullfrogs in this patient, the pleroceroid may have entered the body through the mouth and then entered the liver via the bile duct or portal vein to induce liver injury. However, this case was initially diagnosed as IMCC, and this misdiagnosis could be related to the following two aspects. First, HS is uncommon and has not been previously reported in Eastern China. The physician did not consider the possibility of this disease in the initial diagnosis and did not learn about the patient’s bullfrog-eating habits. Second, the radiological findings of this case of HS are similar to those of IMCC, which, together with the abnormal elevation of CA199 level, finally leads to misdiagnosis.

Intrahepatic cholangiocarcinoma accounts for approximately 15% of primary liver cancers and is the second most common type of primary liver cancer, with IMCC being the most common (9). The typical CT manifestations of IMCC include a hypointense, irregular, and well-defined mass, which shows the “single-target” sign. In other words, the center is a hypointense necrotic region, the marginal capsule wall exhibits ring enhancement, the lesion displays centripetal delayed enhancement, and the delayed enhancement surrounding the lesion shows equidensity at the delayed phase (10). This enhancement mode is related to the histological composition of the tumor, to be specific, the tumor periphery possesses rich active tumor cells, whereas its center is mostly the coagulative necrotic region, with a small amount of cancer cells and interfibrillar substances. Thus, enhancement scanning reveals progressive enhancement and retraction of the liver capsule can also be observed as well. The typical MRI findings of IMCC include hypointensity on T1WI, heterogeneous hyperintensity on T2WI, and target diffusion limitation on DWI (11). Seo et al. (12) suggested that hypointensity on DWI might be due to necrosis and interfibrillar substances in the tumor center, whereas peripheral hyperintensity might represent active tumor cells. In our opinion, among the radiological findings of IMCC, bile duct dilation in the mass periphery, retraction of the adjacent liver capsule, peripheral satellite nodules, and vascular invasion are of certain differentiation value from HS.

CA199 is a type of ganglioside with sialic acid as the main ingredient, isolated from the human colon cancer cell line SW1116 by Koprowski et al. (13) (1979), and is a mucin tumor marker. This marker exists in the serum as mucin and is an important tumor-associated antigen. It is related to the Lewis blood group component, exists in normal tissues and cells, and is not specific to tumor cells (14, 15). Under normal conditions, the serum content of CA199 in normal subjects is less than 37 U/mL, and is primarily distributed in the gallbladder, liver, intestine, pancreas, and bile duct epithelium (16). Some research has discovered that its content is relatively high in digestive system tumors, but relatively lower in the serum of normal subjects. Typically, its content is related to tumor size and is a favorable indicator for detecting digestive malignant tumors (17). However, certain studies have pointed out that CA199 can regulate the migration and adhesion of white blood cells in the inflammatory area to promote the occurrence of white blood cell aggregation; consequently, it is not only a tumor marker but also an inflammatory marker (18). We believe that CA199 may be an awkward tumor marker; when its level is elevated, it may indicate a malignant or benign lesion, which means that it has insufficient diagnostic specificity. Consequently, when making a diagnosis, it is necessary to combine clinical, radiological, and other tumor marker detection results to make a comprehensive judgment.

Sparganosis, especially HS, is rare and there are no definite diagnostic guidelines worldwide; only case reports have been published. Jo et al. (19) reported CT, MRI, and ultrasound imaging findings from one case of HS and discovered that HS manifested as a well-defined, heterogeneous hypoechoic mass on ultrasound examination, with no tumor vessels being observed, whereas CT and MRI findings suggested the presence of a non-enhanced serpentine tubular structure inside the mass, and the diffusion of the DWI sequence was not limited. This patient had no other complaints, and the lesion in the liver was detected during an abdominal CT study performed for the staging of the rectosigmoid colon cancer. Khurana et al. (20) reported a case of a liver abscess induced by Sparganum, in which the patient underwent intrahepatic abscess cavity aspiration, from which polypide was obtained, and the patient completely recovered after anti-parasite treatment. This patient presented to local private hospital with complaints of high fever and vomiting for 4-5 days. Fever episodes were not associated with rash, bleeding or chills and rigor. Haemogram revealed leucocytosis with predominant polymorphonuclear cells and marginally raised eosinophils. Zhao et al. (21) reported a case of HS in the Hunan Province, China. The patient visited the hospital due to abdominal pain, upper abdominal enhanced CT and MRI revealed multiple space-occupying lesions in the liver. Liver needle biopsy pathological examination diagnosed the lesions with granulomatous hepatitis, accompanied by excessive eosinophil infiltration around the granuloma and in the necrotic area. Parasite examinations revealed positivity for pleroceroid antibody IgG and negativity for the rest. Infection with sparganosis was considered; thus, the patient was administered anti-infection and systemic helminthic treatment. Upper abdominal enhanced CT re-examination half a month later revealed that the intrahepatic lesion was markedly smaller than before, routine blood tests were within the normal range, and the abdominal pain symptoms of the patient disappeared. This patient complained of a history of accidental swallowing of river water while swimming a month ago. Xian et al. (22) reported two cases of HS from Guangdong Province, China, with a chief complaint of abdominal pain, which revealed an intrahepatic lesion that disappeared after oral administration of quinolone. Both patients presented with right upper abdominal pain and a history of a raw fish diet. The radiological findings of HS reported in the above literature revealed an intrahepatic lesion; however, the radiological manifestations of the lesion vary greatly, and specific imaging signs are lacking. Considering that the cuticle and metabolites of *S. mansoni* contain allogenic proteins, the human body could develop allergic phenomena and foreign body reactions, and the lesion may be centered on the larva, with an eosinophilic abscess and liquefied necrotic tissue in the periphery (23). As the as-formed tubular structure inside the mass exhibited certain characteristics, the patient reported in this study possessed this radiological sign; however, more cases are warranted for verification. Moreover, in the case of increased peripheral blood eosinophil levels, attention should be paid to the differential diagnosis of parasitic infection; however, the number of eosinophils may not increase in patients with parasitic infection (24).

With changes in lifestyle and dietary habits, the prevalence and clinical manifestations of sparganosis demonstrate new features, and the disease exhibits an increasing incidence rate. The clinical manifestations of HS are non-specific; therefore, the disease is susceptible to misdiagnosis and delayed treatment. Compared with ultrasound and CT examinations, MRI examinations may be more valuable. On upper abdominal MRI-enhanced examination, the stripe-like hypointensity inside the liver lesion is not enhanced, and the sign of progressive enhancement of the lesion margin could provide clues for the diagnosis and differentiation of this disease. Consequently, when space-occupying lesions are discovered in the liver, especially when the peripheral blood eosinophil number increases, apart from the consideration of common and frequently occurring diseases, the possibility of parasite (such as *Sparganum*)-induced liver injury should be excluded in combination with a history of epidemiology, radiology, and immunological examinations to prevent missed diagnoses and misdiagnoses. Patients may have a favorable prognosis if an early diagnosis is made and drug treatment is administered. Optional therapies include praziquantel, albendazole, and mebendazole. Surgical treatment is needed in cases of untimely diagnosis and treatment and the formation of granulomas. Complete removal of the *Sparganum* is the most effective method. The patient in this study received surgical treatment, and no signs of intrahepatic recurrence were observed during the 1-year follow-up period. In addition, prevention of this disease is important. To prevent sparganosis infection, it is necessary to increase publicity, cultivate good eating habits in people, refuse uncooked frogs and snakes, not apply a wound or pustule with frog skin, uncooked frog meat, or snake skin, and utilize multiple health education interventions to realize the effects of prevent and control sparganosis infection. Furthermore, attention should be paid to the organic combination of scientific research with the clinic, and the development of more accurate diagnostic reagents and multiple diagnostic methods, including radiological examinations, should be employed to diagnose sparganosis in a timely manner.

In summary, this case indicates that non-contrast and contrast-enhanced upper abdominal MRI examinations may have certain advantages in diagnosing HS. The possibility of HS should not be ignored when signs such as stripe-like hypointensity inside the liver lesion are not enhanced or when the sign of progressive enhancement of the lesion margin is accompanied by an increased number of eosinophils in the peripheral blood. When diagnosis and treatment are not timely and granulomas have formed, surgical treatment is required, and the prognosis is generally better.

## Data availability statement

The raw data supporting the conclusions of this article will be made available by the authors, without undue reservation.

## Ethics statement

The studies involving humans were approved by Ethics Committee of The Second Affiliated Hospital of Xuzhou Medical University. The studies were conducted in accordance with the local legislation and institutional requirements. The participants provided their written informed consent to participate in this study. Written informed consent was obtained from the individual(s) for the publication of any potentially identifiable images or data included in this article.

## Author contributions

YW: Conceptualization, Investigation, Writing – original draft. YL: Conceptualization, Investigation, Writing – original draft. LC: Conceptualization, Investigation, Writing – original draft. XY: Conceptualization, Software, Writing – original draft. AC: Conceptualization, Data curation, Writing – review & editing. PD: Conceptualization, Funding acquisition, Supervision, Writing – original draft, Writing – review & editing.
